# Analysis of the role of self-efficacy and interpersonal relationships in the relationship between subjective family socio-economic status and college students’ trust character: a case study of a university in Shaanxi Province, China

**DOI:** 10.1038/s41598-025-96358-z

**Published:** 2025-04-04

**Authors:** Ziyue Song, Wenlong Zhao

**Affiliations:** 1 School of Humanities and Social Sciences, Xi’an Jiaotong University, Xi’an, China; 2 School of Educational Sciences, Yan’an University, Yan’an, China

**Keywords:** Subjective family socioeconomic status, Trust character, Self-efficacy, Interpersonal communication, Psychology, Human behaviour

## Abstract

Trust can assist college students in navigating the challenges of loss during their growth and development, ultimately facilitating their personal growth and well-being. From the perspective of virtue, this study proposes a new concept of “trust character”, which reveals the relationship between subjective family socioeconomic status and college students’ trust character through self-efficacy and interpersonal communication. In this study, 1211 college students from a university in Shaanxi Province were surveyed by stratified sampling using the Subjective Family Socioeconomic Status Scale, the Self-Efficacy Scale, the Interpersonal Interaction Scale, and the Trust Character Scale. The results showed that subjective family socioeconomic status was significantly positively correlated with self-efficacy (r = 0.161), interpersonal communication (r = 0.193), and trust character (r = 0.160). Self-efficacy was positively correlated with interpersonal communication (r = 0.461) and trust character (r = 0.616). Interpersonal communication was positively correlated with trust character (r = 0.492). The direct and indirect effects of subjective family socioeconomic status on college students’ trust character were significant, including the mediating value of self-efficacy was 0.080 (61.07%), the mediating value of interpersonal communication was 0.032 (24.43%), and the chain mediating value of self-efficacy and interpersonal communication was 0.019 (effect size) was 14.50%.The research shows that the trust character of college students is composed of three dimensions: trust yourself, trust others, trust people. Self-efficacy and interpersonal communication not only play an independent mediating role but also play a chain mediating role between subjective family socioeconomic status and college students’ trust character.

## Introduction

Trust is the basic ability of social development mastered by individuals in the process of growth and development. For individuals, if they master the pursuit of development by trusting, they can avoid contradictions, conflicts and struggles from within the individual, and then achieve better development. At present, some developed countries, such as the United Kingdo^1^ and Germany^2^, have realized the importance of trust education and incorporated it into school value education programs. Finnish education, which is known as the "world’s first", has been highly evaluated in international assessments such as PISA^3^ for its trust as the cornerstone of the entire education system. With the complex changes in modern society and the continuous expansion of international higher education, it is natural that the issue of trust should become a new issue of educational equity. According to the theory of humanistic development, trust is a kind of character that social people should have, which comes from the unremitting pursuit and exploration of development and creation by social actors. From the perspectives of education, psychology and sociology, the exploration of the influencing factors of trust character is inseparable from the attention to family background.

The socioeconomic status of a family is a core variable in measuring family background. Existing research indicates that family socioeconomic status has a significant positive impact on individual growth and development^4,5^. From a theoretical perspective, both the family stress model and family investment theory provide a framework for understanding the relationship between family socioeconomic status and trust character. These theories suggest that family economic conditions significantly influence students’ growth and development. Families with higher socioeconomic status can invest more human and material resources in their children, which positively affects the formation of trust character. It is noteworthy that recently, academia has distinguished between subjective and objective measurements of family socioeconomic status. With the increasing focus on subjective family socioeconomic status, we believe that exploring the relationship between subjective family socioeconomic status and trust character, as well as its mechanisms, is particularly important.

Self-efficacy is a very important concept in psychology, proposed by Bandura in 1977, and it is a core concept in Bandura’s theoretical system, specifically refers to the degree of confidence that people can use their skills to complete a certain work behavior^6^. In the study of self-efficacy, Bandura proposed the "triadic interaction theory", which emphasizes the role of the external environment and believes that the two-way interaction between the environment, the individual and the behavior is not horizontal and static, but spiraling. Therefore, theoretically, subjective family socioeconomic status, as a basic environmental factor, may have an impact on the individual’s self-efficacy, which determines or influences the individual’s subjective judgment of whether or how to participate in interpersonal communication, and then affects the acquisition of trust character.

Interpersonal communication refers to the activities in which people (actors) who survive and develop in society pass certain information to other individuals through certain means of expression, such as language, writing, body movements, and expressions, so that their ideas can be understood and accepted by others^7^. According to the theory of interpersonal relationships, repeated reciprocal exchange will trigger individual sense of obligation and gratitude, and cultivate trust. On the contrary, the lack of communication will hinder the establishment of trust^8^. As a collective phenomenon, trust points to certain social relations, involving the connection between people and people, people and collectives^9^. In other words, trust originates from the experience and cognition gained by the interaction between individuals and others in the past experience. When people popularize their own experience, the character of trust is formed^10–12^.

In summary, although research on trust has yielded relatively rich results, there remains potential academic space worth exploring in cultivating it as a moral character. Based on this, this study focuses solely on the cultivation of trust character, using Giddens’ structuration theory as a starting point, and approaches it from the perspective of subjective family socioeconomic status, integrating psychological and sociological research on trust, and attempts to propose a "structure-action" analytical framework for examining the construction of trust character among college students. Based on the primary data collected by the authors, this study mainly focuses on the following questions: (1) the connotation of trust character; (2) the relationship between subjective family socioeconomic status and college students’ trust character; (3) the role of self-efficacy and interpersonal communication in the relationship between subjective family socioeconomic status and college students’ trust character. Based on the research findings, this study will offer relevant suggestions to enhance college students’ trustworthiness and encourage universities to pay more attention to shaping trustworthiness in the educational process, promoting individual sustainable development.

## Literature review and research hypothesis

### Trust

From the perspective of virtue, Francis Fukuyama made a corresponding annotation. In the book 《Trust: The Social Virtues and the Creation of Prosperity》 Fukuyama affirmed the primacy of trust as a cultural element in the rise and fall of economic activities^13^. He proposed that trust is the root cause of the gap in economic achievement. A society with a high level of trust can promote economic growth more than a society with a low level of trust. In Fukuyama’s view, trust comes from deep-rooted ethical habits, which belong to the collective habits and moral standards of the group. Like heritage, they are consumed, wasted or accumulated in the slow passage of time unconsciously. If we accept this logic, it means acknowledging that it is difficult to create economic prosperity in societies with low levels of trust. Obviously, such a view has the color of determinism, mechanization of living things, putting the multi-context differences generated by trust on the shelf, and also killing the initiative of the individual. In order to make up for the shortcomings of this logic, Alain Peyrefitte put forward the method of comparative character in the《La societe de confiance》to talk about trust^14^. He believes that the society itself contains the spiritual attribute of trust. It is this spiritual incentive to “develop” that stimulates people’s spirit of innovation, rationality, competition and responsibility. If all the spiritual attributes placed under the banner of trust are removed, everything will be difficult to maintain. Therefore, on the road of development, trust is the power source of social progress. Undoubtedly, Alain Peyrefitte’s research highlights the congenital nature of trust and activates the vitality of trust. Unfortunately, when talking about trust, Alain Peyrefitte’s is more from the social level to find the power source that affects social development, weakening its value expression at the individual level; in addition, his interpretation of trust stays too much at the level of concept, attitude and behavior, thus obscuring the “action” characteristics of trust itself, making it impossible to produce from the facts of character, and then sublimate into a character with exemplary and standard significance.

In view of this, this study introduces the term “practice” and proposes the concept of “trust character”, aiming to deeply engrave this moral behavior standard in the individual’s daily behavior and extend it in the form of character. Here we believe that trust character is composed of trust character and practice. Among them, trust character is a kind of character that social people should have. The characteristics of trust character are to encourage individuals to be responsible, to stimulate competitive interest, and to comply with certain ethics. The understanding of the word “practice” quotes the French sociologist Bourdieu’s view that practice is between habitus and field, which is an unconscious correlation^15^. Bourdieu uses the following equation to summarize this relationship: practice = (habitus) (capital) + field. The equation can be stated as follows: practice is the relationship between a person’s habits and his current situation. From the perspective of the formation basis of trust, trust is naturally contained in each individual, and can be further explored and developed into character through acquired education.

Based on the theory of humanistic development, the author defines trust character as an interdependent and stable moral behavior formed by social actors in the process of social interaction with themselves and others due to the fulfillment of their commitments. This is a concept that integrates value and action, reflecting a person’s confidence in themselves and others. This trust highlights the absolute interdependence between individuals and society, namely trust yourself and trust others, which are two aspects of the same mental movement, both of which are indispensable. Different from previous studies, this view of trust also includes the dimension of self-confidence, which makes up for the shortcomings of previous studies that have neglected the construction of self-relationships. In this sense, self-trust and trust in others together constitute the “knowledge” of the character of trust, making trust the fundamental moral principle that coordinates the relationships between the actor, themselves, and others. However, this trust alone is far from sufficient; the character of trust cannot remain merely at the level of abstract concepts; it must be reflected in the social actions of the actor. This requires the actor to achieve a transformation from “knowledge” to “action” in social interactions, thereby making the character of trust the objective that regulates the relationships between the actor and themselves, as well as with others. Through the practice of social interactions, the actor gradually forms the character of trust by coordinating their relationships with themselves and others based on the principle of trust. As an important moral element, college students’ trust character reflects the following characteristics: first, it exists in the form of relationship, and the object of orientation includes both themselves and others. Secondly, based on the harmony of body and mind, marked by the consistency of words and deeds, and oriented by positive expectations. Thirdly, it is composed of three dimensions: trust yourself, trust others, trust people. Among them, trust yourself comes from trust others’ self-confidence, and the logic of self-confidence is derived from self-identity. Trust others, like the essence of self-confidence, requires a sense of trust to be built in the process of understanding, recognition and acceptance, and trust others is a further expansion of individual self-confidence. Trust people refer to a kind of value recognition of human development.

### Subjective family socioeconomic status and college students’ trust

When studying the issue of trust, the academic community generally believes that the individual’s socio-economic status is the most important factor that cannot be ignored^16–19^. Because trust is accompanied by risk, people need to assess the benefits of being trusted and the losses of being discredited when choosing whether to trust others^20^. Here, groups with higher socio-economic status will have a more open and optimistic attitude towards life because they have more resources. In the face of risks and losses, they have relatively higher affordability and lower “relative vulnerability”^18^, so they will be more inclined to trust others. Bjørnskov found in a multinational study that family socioeconomic status affects educational levels through education, especially in high-income countries^21^. Stamos et al.^22^ also found that Lower childhood socioeconomic status predicts lower levels of trust.Therefore, socioeconomic status is a key factor affecting people’ trust^23–26^.

However, in recent years, Some scholars believe that the objective family socio-economic status constructed by parents’ education level, parents’ occupation and family income has certain limitations, so they gradually shift the focus of research to the subjective family socio-economic status. Subjective family socioeconomic status refers to the individual’s perception of their social class status^27^. In contrast, this measurement breaks the static existence of objective family socioeconomic status, and more comprehensively and profoundly judges the relative position of individuals in society^28^. Because teenagers are at a critical stage of growth and development, they pay more attention to the worship of material and the acquisition of knowledge. They will form a cognition of their social status by comparing with the surrounding groups^29^. Therefore, Glendinning shows that college students are in the stage of transition to the adult world, and their sense of status has been formed, which cannot be simply replaced by objective family socioeconomic status indicators^30^. Looking back at the existing research, we find that the current academic community pays more attention to the impact of subjective inequality on individual development. Based on this, this study proposes the following hypothesis.

#### H1

Subjective family socioeconomic status has a significant positive impact on college students ' trust character.

### The mediating effect of subjective family socioeconomic status on college students’ trust

#### Self-efficacy

Subjective family socioeconomic status is based on objective family socioeconomic status. As an independent structure, it can influence the formation and development of self-efficacy by shaping individual experiences^31^. As a stable state when individuals face social participation, self-efficacy reflects people’s confidence in whether they can use their skills to complete a certain work behavior^6^. Studies have shown that subjective family socioeconomic status can affect individual self-cognition. People with higher socioeconomic status have more positive self-concept and higher self-efficac^32,33^, and also have a positive evaluation of their own ability. Because the higher the family’s socioeconomic status, the more disposable resources the family can provide for their children, and the more respect, understanding and trust in educating children, the higher the family’s socioeconomic status, the higher the children’s self-efficacy and the easier it is to have self-confidence. On the contrary, families with lower socioeconomic status are able to provide less assistance to their children, which can easily lead to conflicts during intergenerational communication, hindering the formation and development of children’s personalities^34,35^. Therefore, it is generally believed that there is a strong correlation between subjective family socioeconomic status and self-efficacy^36–38^.

In addition, self-efficacy can also positively predict the level of trust of college students^39^. Self-efficacy emphasizes the individual’s evaluation of self-ability, which is the necessary psychological condition for the individual to achieve the goal, and affects the result of individual behavior to a certain extend^40,41^. Studies have shown that individuals with high self-efficacy are usually confident in their own abilities, believe that they can achieve success, and are full of expectations for success, which is more conducive to individuals to continuously stimulate their potential and have higher persistence and initiative^42^. When the individual’s evaluation can not meet the certain needs of confidence, it will induce a variety of negative emotions, such as pessimism, cowardice, inferiority and so on. Therefore, self-efficacy is a key factor to enhance self-confidence. People with high self-efficacy are more confident in their own abilities and believe that they can influence the surrounding environment and events. In the face of uncertain situations, they can make more efforts to improve their learning and working environment^43^, thus promoting the formation and development of individual trust.

In view of the connotation and characteristics of college students’ trust character, we speculate that self-efficacy will not only be affected by subjective family socioeconomic status, but also have an impact on trust character. At this point, this study puts forward the following hypotheses.

##### H2

Subjective family socioeconomic status has a positive impact on college students’ trust character through self-efficacy.

#### Interpersonal communication

According to the explanation of college students’ development theory and university influence model, whether college students can better integrate into the two subsystems of academic and social is very important to their growth and development during school^44^, especially the social system. Different from the ascribed family background, the social system of college students belongs to the epigenetic resources. Putnam classifies this social system into two forms, one is a horizontal social network with the same social status and power, and the other is a hierarchical social network composed of people with different levels of social status or power^45^. For college students, their interpersonal communication network is mainly composed of hierarchical teacher-student relationship and horizontal peer relationship. Healthy peer relationship is critical to the positive development of adolescents’ cognitive, emotional, social skills, and scholastic adaptation^46^, which buffer them against the impact of burdensome circumstances in other areas of life^47^. Positive student–teacher relationship is critical to the positive development of adolescents^48^. It provides security, safety, and protection that necessary for students’ full participation in social activities^49^, and supports them in adjusting to school life, improving their social skills and promoting academic achievement^50^. In many studies, we found that the conclusion about the influence of interpersonal communication on trust is very consistent, that is, there is a strong correlation between interpersonal communication and individual trust^51–55^, which shows that a good interpersonal communication system is very important to the construction and promotion of individual trust. In addition, the differences in the status or class of individuals will also be reflected in interpersonal communication and have an impact on trust. Some scholars have found that it is relatively difficult for students from lower-class families to establish contact with peers and teachers^56–58^.Similarly, Chinese scholars have also found that adolescents from families with lower socioeconomic status tend to have relatively poorer quality of social relationships, which includes relationships with parents, peers, and teacher. Among them, the influence of subjective socioeconomic status on peer relationships is slightly stronger, while the impact of maternal education on teacher-student relationships is somewhat stronger^59^. It is evident that the subjective social economic status has a significant impact on the interpersonal communication of college students.

In summary, college students’ interpersonal communication is likely to have a significant mediating effect between subjective family socioeconomic status and college students’ trust character. That is to say, the higher the subjective family socioeconomic status is, the stronger the interpersonal skills of college students are. They are more willing to integrate into collective activities and make friends with teachers and peers. Correspondingly, their trust level is also higher. In view of this, this paper proposes the following hypothesis:

##### H3

Subjective family socioeconomic status has a positive impact on college students’ trust character through interpersonal communication.

#### Self-efficacy and interpersonal communication

Social cognitive theory holds that everyone has a self-system, which provides a reference mechanism and functions of perception, management and evaluation of behavior. These functions come from the interaction between the system and the surrounding environmental resources^60^. According to Bandura’s triadic reciprocal causation theory, there exists a bidirectional interaction among the environment, the individual, and behavior. Therefore, theoretically, interpersonal communication, as a behavioral factor, is influenced by self-efficacy, which is an individual factor. Existing empirical research also indicates that self-efficacy, as a positive psychological factor, has a positive and significant impact on individuals’ interpersonal communication^61^. People with strong self-efficacy can correctly understand themselves and evaluate others. In the process of communication, they often have strong communication motivation and believe that they will be rewarded in communication. In addition, people with strong self-efficacy are more willing to choose to interact with people with excellent performance. However, people with weak self-efficacy are unable to correctly understand themselves and society, and often carry a lot of negative emotions in communication, which greatly hinders interpersonal communication, and even causes communication obstacles in severe cases^62^. It can be seen that self-efficacy can positively predict interpersonal communication, which is no exception for college students. Studies have shown that there is a significant positive correlation between college students’ self-efficacy and interpersonal communication. Enhancing self-efficacy can effectively improve college students’ social communication and collaboration ability.

However, at present, there are few studies on the chain mediating effect of subjective family socioeconomic status on college students’ trust. As the base of individual birth, growth and development, family affects the formation of self-efficacy from the root. Families in the dominant class tend to adopt a positive parenting style, which can promote the generation of individual positive psychology^63^, making them more willing to express their intentions and maintain communication in interaction. With the deepening of interaction, individual trust will be improved. Based on this, this study proposes the following hypotheses.

##### H4

Subjective family economic status indirectly promotes interpersonal skills through self-efficacy, which in turn has a positive impact on college students’ trust character.

## Research framework diagram

The "structure-action" theory was proposed by the British sociologist Giddens based on his critique of dualistic thinking, which concisely and profoundly explains the complex social activities of human beings, and is known for its high degree of balance. This theory holds that the socio-economic structure constitutes the rules and resources of action, and that action is driven and constrained by the social structure, and the two construct each other, emphasizing the historical rootedness of action and social structure and the connection with the spatio-temporal context. This theory has been widely used in the analysis of structural contradictions and dilemmas under various disciplinary themes.

**Fig. 1:**
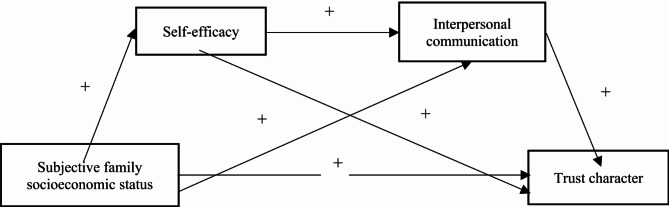
The logical framework diagram of the relationship between subjective family socioeconomic status and the trust character of college students.

In essence, the cultivation of college students’ trust character requires not only the positive action of the subject, but also the support and guarantee of external conditions. Drawing on Giddens’s structuring theory, this study attempts to propose an analytical framework of "structure-action", which combines the macro structure and internal processes of constructing trust character. Specifically, this study involves four core concepts, in which subjective family socioeconomic status represents the individual’s perceived primary social structure, trust character represents the outcome of education, and self-efficacy and interpersonal communication together constitute the whole action of the individual in the university field, and the above concepts together constitute the complete process of "structure-action-outcome". So far, this study proposes a roadmap of the relationship between subjective family socioeconomic status and college students’ trust character (see Fig. 1).

## Materials and methods

### Research data

The empirical data in this study are derived from a questionnaire survey conducted by the research group on undergraduates in a university in Shaanxi Province in December 2023. The main reasons for choosing this university are as follows: First, it is representative of data. Since the university is a comprehensive university, with a complete range of disciplines and a diverse student body, it can provide a rich sampling source for this research. Second, it has data stability. This study was conducted by selecting students from different majors and grades in this university, and these students lived in a relatively stable school environment and institutional environment, which provided an external validity of data stability for this study. Third, it has data feasibility. Due to the researcher’s personal work, he can obtain a very rich amount of data in this university, which is conducive to the conduct of research.

This study has been approved by the Ethics Committee of the School of Humanities of Xi’an Jiaotong University (20231022), and the participants agreed to participate in the study through the informed consent process. The questionnaire covers the basic personal information of college students, the basic situation of the family and the living conditions in school and many other information. This survey adopts the stratified sampling method, in the school students in the proportion of 10% according to the college class stratified sampling, with simple random sampling method to specific classes and students, selected class students all included in the survey sample. This sampling method ensures that the sample is more representative, providing a diverse mix of participants in terms of age, gender, and academic discipline.

The questionnaire survey was completed under the guidance of counselors and class teachers. A total of 1222 questionnaires were collected. After eliminating missing values and invalid answers, 1211 valid samples were entered into the analysis, of which boys accounted for 41.3% and girls accounted for 58.7%. Rural college students accounted for 73.9%, urban college students accounted for 26.1%; grade 1 students accounted for 54.3%, grade 2 students accounted for 20.6%, grade 3 students accounted for 9.4%, grade 4 students accounted for 12.9%, grade 5 students accounted for 2.7%.

### Variables and measurements

#### Dependent variable

**Fig. 2:**
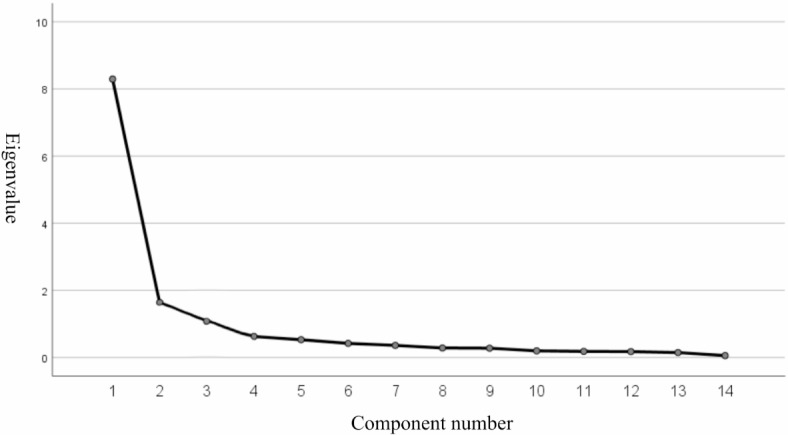
Gravel diagram.

Referencing existing trust scales (Rotter^64^, Rempel and Holmes^65^) and the research by Peyrefitte^14^, and incorporating Chinese local culture, a self-designed college student trust character scale was created, consisting of 14 items all positively scored, using a 1 to 5 point scoring method. According to the pre-test exploratory factor analysis (EFA) of 321 questionnaires, 3 common factors were obtained by combining the main axis factor method with the maximum variance rotation of 14 items. It can be observed from the gravel diagram in Fig. 2 that the first three factors have the highest eigenvalues, after which the curve tends to flatten. The total variance ratio indicates the presence of three common factors with eigenvalues greater than 1 and the cumulative variance contribution rate was 76.856% (see Table 1). The overall reliability of the College Students’ Trust Character Scale was 0.944, and the reliability of trust yourself, trust others, and trust people were 0.828, 0.873, and 0.953 (see Table 2), respectively, which were all greater than the standard of 0.7, indicating that each variable had good internal consistency reliability. The reliability and validity of the questionnaire were also tested by combinatorial reliability (CR) and mean variance extracted (AVE) during confirmatory factor analysis (see Table 3). The CR value of the trust yourself dimension was 0.834 and the AVE value was 0.629, the CR value of the trust others dimension was 0.885 and the AVE value was 0.661, and the CR value and AVE value of the trust people dimension were 0.949 and 0.729. The results indicated that the reliability and validity of the questionnaire were good. Confirmatory factor analysis of formal test data showed good construct validity, with CFI = 0.957, GFI = 0.915, IFI = 0.957, NFI = 0.952, and TLI = 0.946. Finally, the score of college students’ trust character is summed up by 14 items, and a continuous variable with a value of 14–70 is obtained. The higher the score is, the higher the individual’s trust character is.

**Table 1:** Exploratory factor analysis of the trust character scale after rotation component matrix (N = 321).

Dimension	Item	Ingredient			Common factor variance
		1	2	3	
Trust yourself	1. Pursuit of independence, courage to take risks, willing to take responsibility	0.757			0.804
	2. Believe in your own judgment, rather than relying on the judgment of others	0.780			0.726
	3. Welcome competition, and even promote competition	0.799			0.699
Trust others	4. I know what my companion will do; his actions are always predictable to me		0.625		0.695
	5. I have found my companion to be a person who is completely reliable, especially in times of significant events		0.757		0.794
	6. The future is uncertain, but I believe that no matter what happens, my companions will give me strength		0.774		0.812
	7. Most students do not cheat even when they are confident they will not be caught		0.800		0.726
Trust people	8. Believe that people have the ability to untie the bondage, transform the bondage, get rid of the bondage			0.758	0.758
	9. Believe that people can overcome disease and plague			0.771	0.713
	10. Believe that the majority of people in society are trustworthy			0.775	0.789
	11. Believe that everyone contributes to society			0.824	0.721
	12. Believe that everyone has the right to acquire knowledge, knowledge is not the exclusive privilege of			0.855	0.801
	13. Confidence in the ability of people to acquire knowledge and to improve their own destiny contributes to social development			0.878	0.862
	14. When a person enjoys dignity, uses reason, and gives full play to his talents, he moves towards self-realization			0.881	0.859
Sum of squares of rotational loads	Total	5.348	2.790	2.622	
	Variance percentage	38.201	19.927%	18.727%	
	Cumulative percentage	38.201%	58.128%	76.856%

**Table 2:** Reliability test of the trust character scale.

Item		The revised item is related to the total	Cronbach’s Alpha after item deletion	Cronbach’s	Overall reliability
Trust yourself	1	0.687	0.941	0.828	0.944
	2	0.656	0.941		
	3	0.575	0.944		
Trust others	4	0.525	0.946	0.873	
	5	0.729	0.940		
	6	0.765	0.938		
	7	0.737	0.939		
Trust people	8	0.798	0.938	0.953	
	9	0.739	0.939		
	10	0.781	0.938		
	11	0.772	0.938		
	12	0.747	0.939		
	13	0.803	0.938		
	14	0.793	0.938

**Table 3:** Summary table of confirmatory factor analysis for trust character scale.

			Estimate	S.E	C.R	P	Standardization	SMC	CR	AVE
1	< -	F1	1.000				0.872	0.760	0.834	0.629
2	< -	F1	0.884	0.055	16.216	***	0.825	0.681		
3	< -	F1	0.796	0.061	12.991	***	0.668	0.446		
4	< -	F2	1.000				0.621	0.386	0.885	0.661
5	< -	F2	1.185	0.091	13.015	***	0.875	0.766		
6	< -	F2	1.246	0.102	12.247	***	0.898	0.807		
7	< -	F2	1.237	0.106	11.722	***	0.829	0.687		
8	< -	F3	1.000				0.817	0.667	0.949	0.729
9	< -	F3	0.962	0.051	19.014	***	0.766	0.587		
10	< -	F3	1.037	0.055	18.750	***	0.800	0.640		
11	< -	F3	0.953	0.058	16.490	***	0.744	0.553		
12	< -	F3	1.121	0.061	18.422	***	0.883	0.780		
13	< -	F3	1.199	0.060	19.851	***	0.978	0.956		
14	< -	F3	1.162	0.059	19.633	***	0.958	0.918		

*p < 0.05, **p < 0.01,***p < 0.001.

#### Independent variable

1. Subjective family socio-economic status: Referencing the widely used MacArthur Subjective Social Status Scale abroad, in conjunction with the research by Quon and McGrath^66^,the questionnaire measures the subjective family socio-economic status by asking the investigator “Generally speaking, what level do you think your family’s socio-economic status is at”, all of which use a positive scoring format, 1represents the “lower level, 2represents the “lower middle level”, 3represents the “middle level”, 4represents the “upper middle level”, and 5represents the “upper level”. The higher the score, the higher the subjective family socio-economic status.
2. Self-efficacy: The General Self-Efficacy Scale^67^ (GSES), which is widely used in the world, is used to measure self-efficacy in the questionnaire. The scale was developed by German psychologist Schwarzer (1997) and his colleagues. There are 10 items in the scale, all of which use a positive scoring format, and each item corresponds to four options. 1 represents “completely inconsistent”, 2 represents “not quite consistent”, 3 represents “relatively consistent”, and 4 represents “completely consistent”. Finally, the score of self-efficacy is summed up by 10 items, and the value range is 10–40 points. The Cronbach 's α of the scale was 0.958.
3. Interpersonal communication: Using questions related to interpersonal interactions from the Chinese College Student Survey^68^ (CCSS) questionnaire for measurement. The interpersonal communication in this study is composed of peer interaction and teacher-student interaction. Among them, peer interaction is measured by asking the frequency of students and peers doing something together, including “self-study with peers”, “discussing academic problems with peers”, “telling hearts with peers”, and “relaxing with peers”. Teacher-student interaction is measured by asking the frequency of interaction between students and teachers, including “discussing topics, ideas or concepts in the course with teachers”, “discussing their own career plans and ideas with teachers”, “discussing world outlook, outlook on life and values with teachers”, “discussing work outside the course with teachers (such as student unions, associations, etc.)”. All of which use a positive scoring format. Each question corresponds to five options, 1 represents “never”, 2 represents “rarely”, 3 represents “sometimes”, 4 represents “often”, and 5 represents “always”. The score of interpersonal communication is summed up by eight items, and the value range is 8–40 points. The higher the score, the higher the intensity of interpersonal communication. The Cronbach’s α of the scale was 0.887.

#### Control variables

Considering that other factors may have an impact on trust, this study controlled variables such as gender, grade, household registration^69^ campus culture atmosphere^70^, and academic performance^71,72^. Gender is coded as: male = 0, female = 1. Household registration is a binary variable: agricultural = 0, non-agricultural = 1. Grade is coded as: first grade = 1, second grade = 2, third grade = 3, fourth grade = 4, fifth grade = 5. Campus culture atmosphere and academic performance are both divided into five levels, with 1, 2, 3, 4, and 5 points from low to high.

### Analytical strategy

In order to comprehensively analyze the relationship between subjective family socioeconomic status and college students’ trust character, SPSS26.0 software package and AMOS24.0 software were used for data processing analysis. Firstly, the Harman single-factor method is used to test for common method bias. A non-rotating exploratory factor analysis is conducted on all variables to examine whether common method bias exists in this study. Secondly, in the case of controlling variables, the degree of correlation between the independent and dependent variables is described and tested by performing partial correlation analysis on the two variables. Thirdly, under the condition of controlling for other variables, a multiple linear regression model was used to investigate the relationship between subjective family socioeconomic status, self-efficacy, interpersonal communication and college students’ trust character. All three of these steps are performed in SPSS26.0software package. Fourthly, a structural equation model was constructed by using AMOS24.0 software to test the overall model fit degree of the path relationship between self-efficacy and interpersonal communication between subjective family socioeconomic status and trust character. The ratio of chi-square to degrees of freedom (χ^2^/df), GFI, AGFI, CFI, TLI and RMSEA were used as evaluation reference values to evaluate and modify the overall model of this study. Fifthly, the significance of the mediation effect was tested using the Bootstrap method. The Bootstrap test estimates the mediating effect by repeated sampling from the sample data, and establishes a confidence interval, which indicates that the mediating effect is significant when the confidence interval does not contain 0.The mediation analysis method is superior to the traditional Baron and Kenny’s stepwise test and Sobel test, which can not only consider multiple mediating variables in one model at the same time, but also obtain more accurate test results without satisfying the normal distribution. This method can provide a clearer understanding of the path relationship between subjective family socioeconomic status and college students’ trust character. This step is also performed in AMOS24.0 software.

## Results

### Common method bias test

In order to avoid the influence of common method bias on the research results, this study employed the Harman single-factor test. All variables were subjected to non-rotating exploratory factor analysis. The results showed that the coefficient of variation of the maximum eigenvalue was 11.71%, which was less than the critical value of 40%, indicating that there was no serious common method bias in this study.

### Descriptive statistics and correlation analysis of subjective family socioeconomic status, self-efficacy, interpersonal communication and trust character

After controlling variables such as gender, grade, household registration, school culture atmosphere and academic performance, this study used partial correlation analysis to explore the Pearson correlation coefficient between family socioeconomic status, self-efficacy, interpersonal communication and trust character. It can be seen from Table 4 that there is a weak or moderate significant positive correlation between several variables, and the relationships between each independent variable and the trust character are all independent.

**Table 4:** Descriptive statistical analysis and correlation of each variable (N = 1211).

Variable	M ± SD	1	2	3	4	5	6	7	8	9
1. Gender	–	1								
2. Grade	–	− 0.035	1							
3. Household registration	–	0.013	0.023	1						
4. School cultural atmosphere	3.71 ± 0.95	0.074**	− 0.02	0.085**	1					
5. Academic Achievements	2.99 ± 0.90	0.108***	0.052	0.052	0.056*	1				
6. Subjective family socio-economic status	2.28 ± 0.79	0.115***	− 0.058*	0.191***	0.114***	0.203***	1			
7. Self-efficacy	25.69 ± 0.64	− 0.061*	0.035	0.139***	0.246***	0.224***	0.161***	1		
8. Interpersonal communication	25.60 ± 0.61	0.032	0.012	0.085**	0.408***	0.227***	0.193***	0.461***	1	
9. Trust character	50.45 ± 9.89	0.017	− 0.001	0.108***	0.354***	0.189***	0.160***	0.616***	0.492***	1

*p < 0.05, **p < 0.01, ***p < 0.001.

### The relationship between subjective family socioeconomic status and college students’ trust character: a dual mediation test of self-efficacy and interpersonal communication

In order to explore the predictive relationship between subjective family socioeconomic status, self-efficacy and interpersonal communication on trust character, on the basis of correlation analysis, this study conducts multiple linear regression analysis on subjective family socioeconomic status, self-efficacy and interpersonal communication, and explores their relationship with trust character(see Table 5).

**Table 5:** Regression analysis results of subjective family socioeconomic status, self-efficacy and interpersonal communication on trust character.

Model		Overall fitting coefficient			*β*	SE	t	Sig	Tolerance	VIF
		*R*	*R*^*2*^	*F*						
1	(Constant)					1.707	17.319	0.000		
	Gender				− 0.035	0.536	− 1.310	0.190	0.975	1.026
	Grade				0.000	0.222	− 0.005	0.996	0.990	1.010
	Household registration				0.056	0.606	2.101	0.036	0.958	1.044
	School cultural atmosphere				0.333	0.277	12.525	0.000	0.978	1.023
	Academic Achievements				0.154	0.298	5.690	0.000	0.946	1.057
	Subjective family SES	0.408	0.166	39.979	0.084	0.347	3.056	0.002	0.907	1.103
2	(Constant)					1.508	11.497	0.000		
	Gender				0.026	0.447	1.155	0.248	0.961	1.040
	Grade				− 0.016	0.184	− 0.716	0.474	0.989	1.011
	Household registration				0.005	0.504	0.243	0.808	0.949	1.054
	School cultural atmosphere				0.209	0.236	9.228	0.000	0.925	1.081
	Academic Achievements				0.045	0.252	1.955	0.051	0.907	1.102
	Subjective family SES				0.034	0.289	1.475	0.141	0.899	1.113
	Self− efficacy	0.654	0.428	128.480	0.550	0.036	23.441	0.000	0.865	1.157
3	(Constant)					1.656	13.798	0.000		
	Gender				− 0.024	0.497	− 0.954	0.340	0.974	1.027
	Grade				− 0.006	0.206	− 0.251	0.802	0.989	1.011
	Household registration				0.048	0.562	1.938	0.053	0.958	1.044
	School cultural atmosphere				0.184	0.281	6.641	0.000	0.824	1.214
	Academic Achievements				0.083	0.282	3.242	0.001	0.909	1.100
	Subjective family SES				0.041	0.324	1.593	0.111	0.894	1.119
	interpersonal communication	0.531	0.282	67.632	0.387	0.045	13.961	0.000	0.778	1.285
4	(Constant)					1.494	10.313			
	Gender				0.024	0.436	1.098	0.273	0.961	1.040
	Grade				− 0.017	0.180	− 0.787	0.431	0.989	1.011
	Household registration				0.008	0.493	0.353	0.724	0.949	1.054
	School cultural atmosphere				0.148	0.245	6.270	0.000	0.818	1.222
	Academic Achievements				0.022	0.249	0.968	0.333	0.891	1.122
	Subjective family SES				0.018	0.283	0.792	0.428	0.891	1.122
	Self− efficacy				0.480	0.038	19.490	0.000	0.748	1.337
	interpersonal communication	0.674	0.455	125.294	0.201	0.042	7.732	0.000	0.673	1.486

It can be seen from Model 1 that the higher the subjective family socioeconomic status is, the higher the trust character of college students is. The subjective family socioeconomic status has a significant positive impact on the trust character of college students, and Hypothesis 1 is established. Comparing the results of Model 2 and Model 1, it can be seen that self-efficacy has an independent influence on college students’ trust character. The higher the self-efficacy, the higher the college students’ trust character. Comparing Model 3 and Model 1, we can find a similar conclusion, that is, interpersonal communication has an independent influence on college students’ trust character. The richer the interpersonal communication is, the higher the college students’ trust character is. In model 4, self-efficacy and interpersonal communication jointly improved the explanatory power of college students’ trust character by 28.9%, and the explanatory power of the whole model reached 45.5%. It should be noted that the standardized regression coefficient corresponding to the subjective family socioeconomic status gradually decreases with the addition of self-efficacy and interpersonal communication variables, and the significance also disappears. In view of this, we speculate that subjective family socioeconomic status is likely to have an impact on college students’ trust character through self-efficacy and interpersonal communication.

**Fig. 3:**
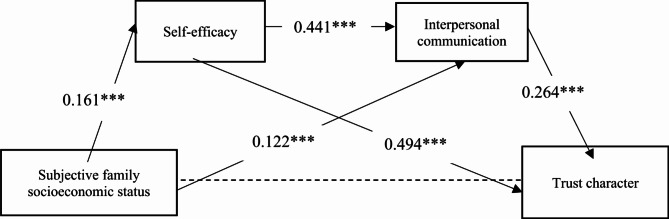
The relationship model between subjective family socioeconomic status and trust character. *p < 0.05, **p < 0.01,*** < 0.001; χ^2^/df = 1.991, GFI = 0.999, AGFI = 0.992, CFI = 0.999, TLI = 0.994, RMSEA = 0.029.

**Fig. 4:**
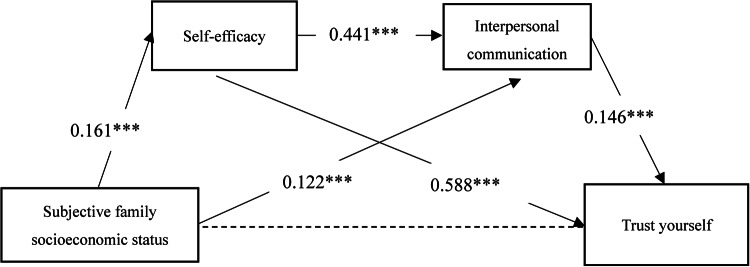
The relationship model between subjective family socioeconomic status and trust yourself. *p < 0.05, **p < 0.01,*** < 0.001.

**Fig. 5:**
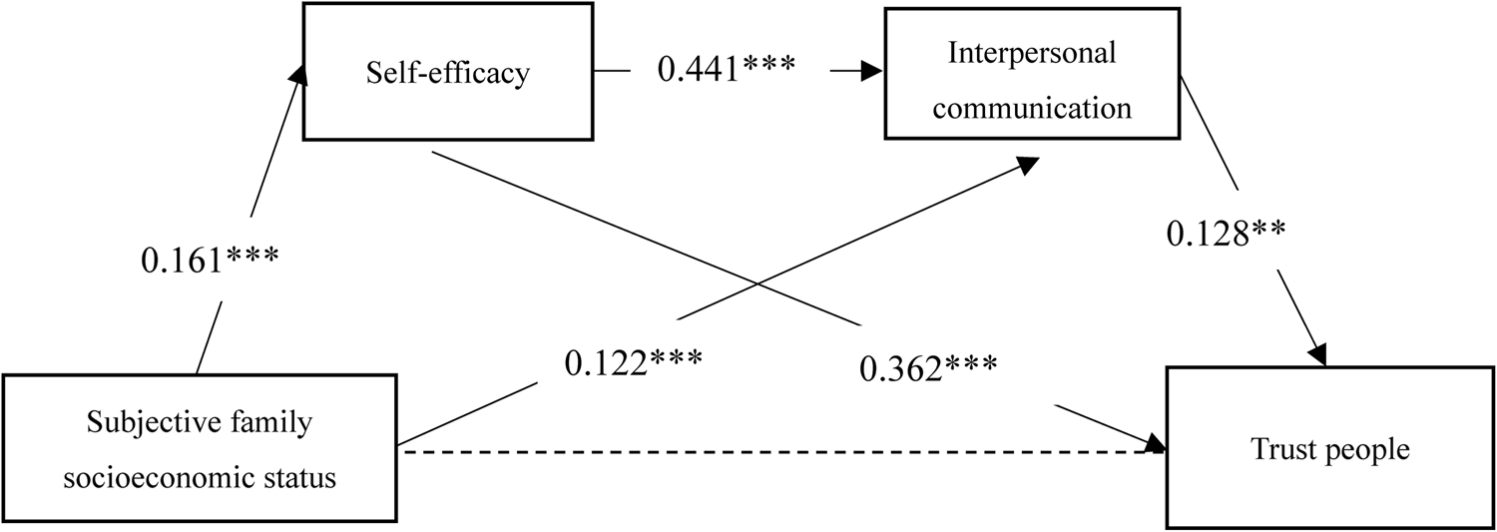
The relationship model between subjective family socioeconomic status and trust others. *p < 0.05, **p < 0.01,*** < 0.001.

**Fig. 6:**
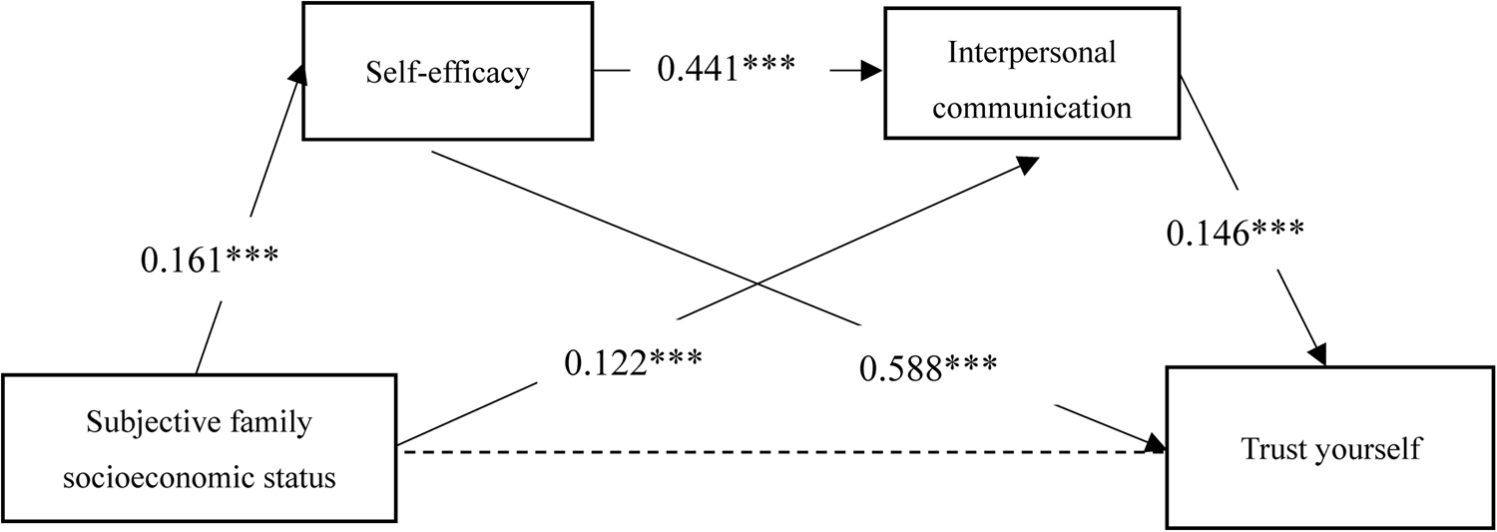
The relationship model between subjective family socioeconomic status and trust people. *p < 0.05, **p < 0.01,*** < 0.001.

Next, this study used AMOS24.0 software to build a structural equation model and conducted a path analysis on how subjective family socioeconomic status affects college students’ trust character. After removing the relationship between subjective family socioeconomic status and trust character, the final results of the model fit test were obtained. In the overall model of this study, the chi-square to degrees of freedom ratio (χ^2^/df) was 1.991, with GFI, AGFI, CFI, and TLI all greater than 0.90, and RMSEA less than 0.05, indicating a good model fit. Figure 3 shows the standardized path coefficients of the impact of subjective family socioeconomic status on trust character. The results indicate that subjective family socioeconomic status significantly positively predicts self-efficacy (*β* = 0.161, *p* < 0.001) and significantly positively predicts interpersonal communication (*β* = 0.122, *p* < 0.001); self-efficacy significantly positively predicts interpersonal communication (*β* = 0.441, *p* < 0.001) and significantly positively predicts trust character (*β* = 0.494, *p* < 0.001); interpersonal communication significantly positively predicts trust character (*β* = 0.264, *p* < 0.001). Figures 4, 5, and 6 show the relationships and influence paths between subjective family socioeconomic status and the three sub-dimensions of trust character.

Finally, Bootstrap was used to test the significance of the mediation effect, and the Bootstrap was set to sample 2000 times, and the confidence level of the confidence interval was 95%. The results showed that subjective family socioeconomic status could have an impact on trust character through three paths(See Table 6): the first path was through self-efficacy, with a mediating effect of 61.07% (0.080/0.131), the second path was through interpersonal communication, with a mediating effect of 24.43% (0.032/0.131), and the third path was through self-efficacy and interpersonal communication, with a mediating effect of 14.50% (0.019/0.131).

**Table 6:** Bootstrap analysis of the significance test of mediating effect and its effect size.

Influence path	Standardized coefficient	Relative mediating effect (%)
Subjective family status → Self-efficacy → Trust character	0.080	61.07
Subjective family status → Interpersonal communication → Trust character	0.032	24.43
Subjective family status → Self-efficacy → Interpersonal communication-trust character	0.019	14.50
Total Indirect effect	0.131	100

## Discussion

As an important moral element, trust can awaken the rational consciousness and deep values of human development, enabling individuals to become better versions of themselves. However, while current research on trust has yielded substantial results in fields such as psychology, economics, and sociology, it is relatively rare in the field of education. Given the significance of trust for individual development, this study begins by pointing out the logical flaws in Fukuyama’s theory of the legacy of trust, then affirms Peretti’s approach from the perspective of character studies to address the shortcomings of Fukuyama’s research. It subsequently supplements the lack of action significance of trust from the character studies perspective using Bourdieu’s practice theory. Finally, based on human-centered development theory, it integrates Peretti’s trust theory and Bourdieu’s practice theory, proposing a new concept of “trust character” and operationalizing it. Compared with previous studies, this research’s understanding of the dimension of trust includes not only trust in others and trust in society, but also trust in oneself, and posits that self-confidence and trust in others are the same movement of the mind, together constituting trust. Based on the author’s primary survey data, this study follows a "structure-action-outcome" logical framework, using subjective family socioeconomic status as a starting point, integrating perspectives from psychology and sociology, with self-efficacy and interpersonal communication as mediating variables. It employs various empirical methods to analyze the relationship and pathways between subjective family socioeconomic status and college students’ trust character.

The study found that: First, the trust character of college students exists in urban and rural areas, and the trust character of urban college students is significantly higher than that of rural college students. Influenced by the long-term urban–rural dual division, the allocation of urban and rural educational resources in China is not balanced, and high-quality educational resources are often concentrated in cities. Rural students entering cities from rural areas will inevitably experience “cultural shock” and “mentality adjustment”. For rural students, leaving home to enter the school is not only the movement of life coordinates in geographical location, but also the attribute of “upward climbing” and “class crossing” in the sociological sense. As a special cultural institution, schools have the social function of helping individuals acquire knowledge, skills and value norms, and the values and beliefs they believe in are often cherished by urban elite groups. Therefore, the difference between rural college students and urban college students in trust character is easy to produce^69^. Second, academic performance can significantly predict the trust character of college students. As an important indicator of evaluating students’ growth and success, academic performance has a non-negligible impact on college students’ current learning life and future job development. If their academic performance is excellent, it means that they have strong professional ability and can take advantage of the awards, so as to gain academic self-confidence. This self-confidence can also arouse the trust of others, and then react on themselves to form a benign cycle^71,72^. Third, the school cultural atmosphere has a significant positive impact on the formation of college students’ trust character. Studies have also confirmed that in organizations with a strong culture of trust, such as close families, friendly groups, etc., will be conducive to the members of the organization to produce “trust impulse” and gradually established in the personality. Therefore, creating a good campus cultural atmosphere can build a legalized institutional environment for the cultivation of college students’ trust character. Under the infiltration of this culture, students will cherish the value of trust and be willing to take action^70^. Fourthly, after controlling the above variables, the sense of status formed by college students subjectively based on how much family resources they occupy will still have a significant impact on their trust character. Hypothesis 1 is validated. Consistent with previous studies, individuals with higher subjective family socioeconomic status had higher levels of trust^73,74^. In addition, it is found that the direct relationship between subjective family socioeconomic status and college students’ trust character is not significant after the inclusion of two mediating variables, self-efficacy and interpersonal communication, suggesting that the relationship between the two may play a role through the mediating variables.

In order to further explore the relationship between subjective family socioeconomic status and the trust character of college students, this study employed structural equation modeling to conduct a mediation effect test, and we have the following findings: First, self-efficacy plays a mediating role between subjective family socioeconomic status and college students’ trust character, and Hypothesis 2 has been validated. According to social comparison theory, individuals with a higher subjective family socioeconomic status have a more positive perception of their own status and are more likely to make positive evaluations of themselves^34,35,75^, thereby achieving a higher level of self-efficacy. Individuals with high self-efficacy believe that they are capable of completing tasks and have strong self-confidence^42^. At the same time, this resilience bias makes them more willing to take risks and choose to trust others, and if they are faced with the mistakes of others, they will also attribute them to situational rather than personal trait defects, and this mode of explanation increases positive judgments about others^61^. Therefore, the stronger the self-efficacy, the higher the individual’s level of trust character. In contrast, individuals with low subjective social status often have a more negative self-evaluation^76,77^.

Secondly, interpersonal communication serves as a mediating factor between subjective family socioeconomic status and college students’ trust character, and Hypothesis 3 has been validated. This result is consistent with previous research findings, indicating that family socioeconomic status not only influences the development of individual cognition, emotions, and personality^78^ but also aids in establishing healthy social networks^34^. Compared to those in lower subjective social classes, individuals in higher subjective social classes are more willing to cooperate and share resources in interpersonal relationships. Through self-disclosure that progresses from shallow to deep, they form a progressive chain of 'information exchange—emotional resonance—value alignment’ in their interactions, which significantly increases the efficiency of trust building^51^. This indicates that interpersonal communication is not only influenced by subjective family socioeconomic status but also promotes individual trust^54,55^.

Thirdly, self-efficacy and interpersonal communication play a chain mediating role between subjective family socioeconomic status and college students’ trust character, and Hypothesis 4 has been validated. This provides a new perspective for understanding the relationship between subjective family socioeconomic status and trust character. Firstly, self-efficacy can significantly and positively predict interpersonal communication. Individuals with higher self-efficacy are more confident in their interpersonal interactions; they are more likely to proactively connect with others, and when faced with challenges and stress, they are more likely to adopt positive coping strategies and exhibit more generous and altruistic behaviors rather than avoidance or negative responses^79^. Therefore, enhancing self-efficacy can improve individuals’ interpersonal communication abilities and reduce social anxiety, which helps them maintain a positive and constructive attitude in their interactions. Secondly, the higher an individual’s subjective family socioeconomic status, the more they perceive themselves to be in a superior environment with richer resources, leading to a greater sense of self-worth. When their self-worth is stronger, their self-recognition is also higher, which in turn enables them to better perceive the needs of others and provide assistance. Through repeated interactions, the character of trust gradually forms.

### Theoretical implications

The theoretical contributions of this study are as follows: First, based on the misunderstanding of trust by Fukuyama and Alain Perrefit, a new concept of “trust character” is proposed, which broadens the perspective of trust research. Second, this paper breaks through the previous research that focuses too much on the measurement of objective family socioeconomic status, turns the attention to subjective family socioeconomic status, and analyzes the relationship between it and college students’ trust character. Thirdly, the attention of comprehensive psychology on psychological traits and the attention of sociology on interpersonal interaction are used to analyze and compare the impact of psychological traits on college students’ trust character, and to further test whether they play a mediating role in the relationship between family socioeconomic status and trust character.

### Practical implications

The greatest practical significance of this study lies in providing a new approach for educators in higher education in the new era, especially for those working with students, to cultivate moral integrity. On the issue of “cultivating what kind of people”, taking the trust character of college students as the starting point, the education work has achieved a real sense of soft landing, making higher education radiate new vitality. In the face of the improvement of college students’ trust character, this study provides some possible ideas and paths. First of all, college educators need to realize that with the continuous expansion of higher education, the inequality of higher education is not only reflected in the enrollment opportunities, but also in the whole process of higher education. As a measure of college students’ family background indicators, subjective family socioeconomic status and household registration still affect the amount of trust character of college students. College educators need to give more resources and platform support to college students with family background in vulnerable groups. Secondly, college educators should screen out vulnerable groups of students according to the actual situation, strengthen classified guidance according to the characteristics of students’ development at different stages, reasonably set up different grades and different groups of education methods, and ensure that education work runs through all stages of college students from enrollment to graduation, so as to realize “all-responsible” and “all-time” education. Thirdly, improving self-efficacy and encouraging students to communicate with each other are helpful to the formation of college students’ trust character. The positive psychology represented by self-efficacy has a continuous and stable influence on trust character. Colleges and universities can integrate trust education into classroom teaching, and encourage college students with disadvantaged family socio-economic status to be optimistic, diligent and self-reliant. In addition, colleges and universities can also explore some special courses that can effectively stimulate college students’ interpersonal communication or social skills, and help college students establish a good teacher-student relationship and peer relationship. Finally, a good campus cultural atmosphere can effectively promote the improvement of college students’ trust character. College educators should continue to make efforts in the construction of campus culture, and create a positive, equal, friendly and trustworthy campus cultural atmosphere by carrying out rich and colorful cultural activities.

### Limitations and prospects

The present study has several limitations: first, due to the limitation of funding and manpower, the questionnaire survey only involves one university, and the data source is too single, so the sample scope should be expanded in the future to verify the generalizability of the results. Second, this study is a cross-sectional study, and the causal relationship between the research variables cannot be clarified, and it can be explored in depth through tracking data in the future. Thirdly, a good school atmosphere and social atmosphere can promote the development of college students’ trust character, and the influence of school and society on college students’ trust character can be deeply explored in the future.

### Conclusions

This study finds that: First, college students’ trust character includes three dimensions: trust yourself, trust others and trust people. Second, subjective family socioeconomic status has a significant positive impact on trust character. Thirdly, self-efficacy and interpersonal communication play an independent mediating role and a chain mediating role between subjective family socioeconomic status and trust character.

## Data Availability

The datasets used and/or analysed during the current study available from the corresponding author on reasonable request.
